# Development of a systematic coarse-grained model for poly(caprolactone) in melt

**DOI:** 10.12688/openreseurope.21354.1

**Published:** 2025-09-23

**Authors:** Petra Bačová, Gonzalo González Huarte, Vagelis Harmandaris, Sergio I. Molina

**Affiliations:** 1 Departamento de Ciencia de los Materiales e Ingeniería Metalúrgica y Química Inorgánica, Facultad de Ciencias, IMEYMAT, Universidad de Cadiz - Campus de Puerto Real, Puerto Real, 11510, Spain; 2 Computation-based Science and Technology Research Center, The Cyprus Institute, 20 Constantinou Kavafi Str., Nicosia, 2121, Cyprus; 3 Institute of Applied and Computational Mathematics (IACM), Foundation for Research and Technology Hellas (FORTH), Heraklion, GR-70013, Greece; 4 Department of Mathematics and Applied Mathematics, University of Crete, Heraklion, GR-71409, Greece

**Keywords:** molecular dynamics simulations, polymer physics, systematic coarse-graining, atomistic model, poly(caprolactone)

## Abstract

**Background:**

This study introduces a systematic coarse-graining approach to model poly(ε-caprolactone) (PCL) in its melt state. The primary goal is to provide a simple and adaptable method for creating computational models of biodegradable polymers, which can then be used to study materials with a wide range of molecular weights and compositions that are relevant to industry. This research addresses the growing need for sustainable materials across various industrial applications.

**Methods:**

To study long polymer chains, the L-OPLS force field, an adapted version of the OPLS-AA force field, was used for atomistic simulations. The data from these simulations were first thoroughly checked against existing literature and theoretical predictions to ensure their validity. These validated atomistic configurations then became the foundation for developing the coarse-grained model.

**Results:**

The research meticulously measured both the structural and dynamic properties of the PCL at the atomistic and coarse-grained levels. The findings show that the model is successful at accurately reproducing key characteristics across these different levels of resolution.

**Conclusions:**

The methodology presented in this work aims to facilitate the development of computational studies that can help optimize the properties of PCL-based materials. By doing so, it has the potential to reduce the environmental and economic impact of developing new sustainable materials.

## Plain Language Summary

This article describes the development and validation of a simplified computational model for a biodegradable polymer called poly(ε-caprolactone), or PCL. PCL is a sustainable alternative to traditional plastics, but simulating its properties for industrially relevant applications, such as in tissue engineering and 3D printing, can be very computationally demanding.

To overcome these computational challenges, a "coarse-grained" (CG) model was developed. This modeling approach simplifies the system by lumping groups of chemically bonded atoms into single beads, which significantly improves computational efficiency while still capturing the material's essential properties. The model was built using a "bottom-up" methodology, where data from more detailed, fine-grained (atomistic) simulations were systematically used to derive the parameters for the reduced-resolution model.

The study found that the coarse-grained model was very successful at reproducing the structural and dynamic properties of PCL. This new methodology is intended to facilitate computational studies, which can help optimize the properties of PCL-based materials and reduce the economic and environmental impact of developing new sustainable materials.

## Introduction

In today’s society, industries have extremely high demands that force them to mass produce to meet these needs. In the manufacturing process, industries mostly opt for the use of plastic due to its wide variety of applications, durability and cost. However, the depletion of oil resources along with increasing environmental pollution have resulted in a global concern that has led to the search for another more sustainable approach for the environment ^1^.

Poly( *ε*-caprolactone) (PCL) is a widely recognized and extensively researched biodegradable polymer, highly valued for its biocompatibility and degradability under physiological conditions ^2^. Derived from petroleum, PCL is a semicrystalline and hydrophobic material, with properties such as crystallinity and biocompatibility highly dependent on its molecular weight ^3^. It possesses a unique combination of characteristics, including high solubility, a low melting point and glass transition temperature, and high compatibility in mixtures, making it a material with significant potential across various fields and applications, such as tissue engineering ^2^ and additive manufacturing ^4, 5^.

A fundamental understanding of PCL’s properties at the molecular level is crucial for optimizing its performance in these advanced applications. While atomistic molecular dynamics (MD) simulations are powerful tools for investigating the dynamic evolution of molecular systems and condensed matter, they are often limited by accessible length and time scales. Simulating complex polymer-based materials, especially high molecular-weight entangled chains over industrially relevant scales, presents a significant computational challenge due to the computational cost and time constraints.

To overcome these computational limitations, multiscale modeling approaches are increasingly employed ^6– 8^. Specifically, systematic coarse-grained (CG) models are developed to reduce the resolution of the system by lumping groups of chemically bonded atoms into single beads. This allows for the simulation of larger systems over extended time periods, significantly improving computational efficiency while still capturing essential material properties ^7^. The development of such CG models often follows a “bottom-up” approach, where information from more detailed, fine-grained (atomistic) simulations is systematically used to derive the parameters for the reduced-resolution models. This methodology typically involves two key steps for building a CG model: i) defining the CG mapping: This establishes the correspondence between the atomistic and the reduced resolutions, determining how atoms are grouped into CG beads; ii) defining the CG effective potentials: This involves deriving the interaction potentials for the CG beads based on the statistics obtained from the atomistic system. Techniques such as iterative Boltzmann inversion (IBI) ^9^ are commonly utilized to match the local structural distributions of the CG model to those of the atomistic model. This ensures that the CG model accurately reproduces the structural and, with appropriate time scaling, the dynamical properties of the underlying atomistic system.

This systematic linking of simulation methodologies across different scales is essential for quantitative prediction of polymer behavior over broad spatiotemporal scales. For PCL, all-atom molecular dynamics simulations, often employing force fields like the Optimized Potentials for Liquid Simulations All-Atom (OPLS-AA) force field ^10– 12^, have been used to study fundamental properties such as melt density, transition temperatures and mechanical characteristics ^13– 15^. Yungerman *et al.*, utilized the OPLS-AA and Class II Polymer Consistent Force Field (PCFF) to investigate the properties of telechelic PCL with diacrylates as reactive functionalities ^15^. Their results indicated that both PCFF and OPLS-AA were capable of describing satisfactorily the geometry of individual PCL chains in dilute and melt systems, and both force fields provided properties such as density and Young’s modulus comparable to the experimental data. The articles by Di Pasquale *et al.* primarily employed the OPLS-AA force field in their investigation of PCL chains consisting of up to 30 repeating units within various solvent environments, specifically water-acetone mixtures ^16, 17^. Their research aimed to understand the precipitation of polymer nanoparticles and the complex influence of solvent composition on the PCL chain configuration. Ezquerro *et al.* chose OPLS-AA force field over AMBER force field to model polymer melt due to its superior performance in predicting PCL’s density ^14^. The research specifically investigated neat PCL polymer matrices of relatively short chains containing 20 monomers, which were then blended into nanocomposite systems containing either ungrafted silica nanoparticles or silica nanoparticles grafted with polyethylene glycol. Recently, Nie *et al.* introduced a priori temperature rescaling method (Hybrid Iterative Boltzmann Inversion - HIBI) for coarse-graining linear PCL chains in melt with the molecular weight of 5700 g/mol ^6^. The method aims to compensate for the loss of entropy by scaling up the temperature a priori and successfully reproduces structural distributions, heat capacity, and Young’s modulus, however, its prediction for viscosity falls short compared to the all-atom systems ^6^.

The current work has as its primary objective to develop a reliable and relatively simple systematic coarse-grained model for linear PCL with the capability of being extended to experimentally relevant time and length scales. We based the model on all-atom simulations of PCL which employed OPLS-AA force field adapted for studying long chains. The atomistic data were first compared to the literature data and then the polymer chains were coarse-grained at the monomeric level. The IBI method was used to capture the structural features of the studied melts and the obtained potentials were employed to simulate CG systems.

A fundamental novelty of this work resided in the investigation of PCL chains across a wide range of molecular weights, specifically from 10 up to 125 monomers (equivalent to 1143 to 14270 g/mol). This expanded range, covering the molecular weight regime from unentangled (Rouse-like) to mildly-entangled systems, provides a more robust platform for studying the influence of chain length effects on fundamental structural and dynamical properties of PCL. Study of the chain length effects is particularly relevant for industrial applications, such as additive manufacturing (3D printing), where properties like rheological behavior (e.g., viscosity) are affected by the degree of crystallinity and thus by the molecular weight of the polymer melt. The ultimate goal is to facilitate the development of the computational approaches, which would speed up the process of tuning the properties of PCL-based materials and by doing so reducing the ecological and economical impact of the property optimization of these new generation of materials.

## Methods and simulation details

**Figure 1.**
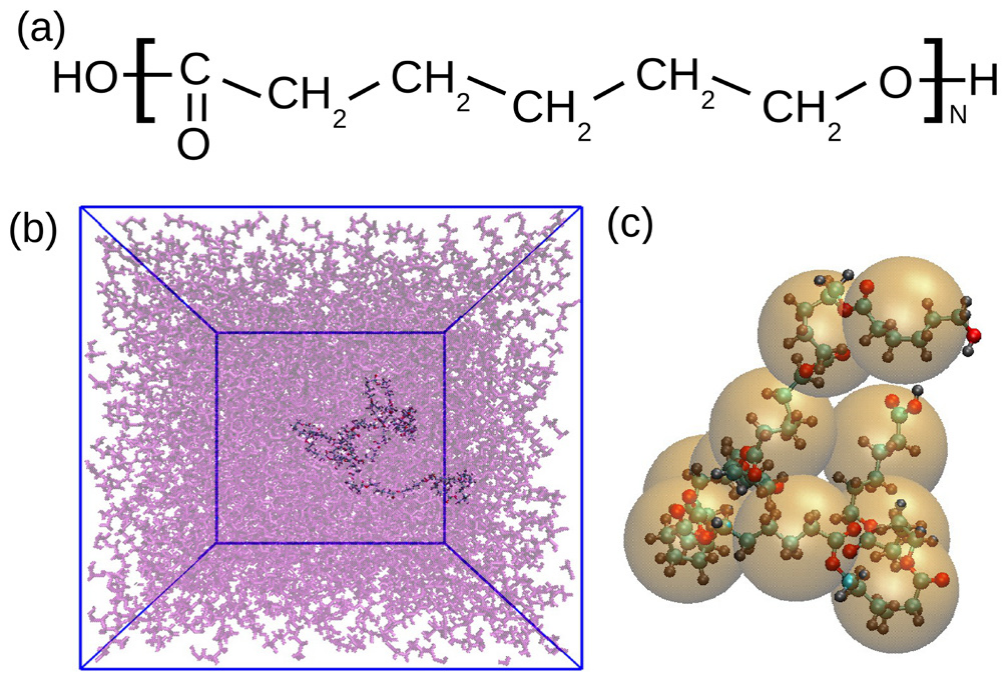
( **a**) Chemical structure of PCL. ( **b**) Snapshot of the PCL chain of 30 monomers in melt. One chain is highlighted while the surrounding molecules are transparent for a better visibility. ( **c**) Schematic illustration of the coarse-graining procedure for PCL10. Each orange sphere represent one CG unit placed in the center of mass of a monomer.

In our study we employed all-atom molecular dynamics simulations to study linear chains of poly(caprolactone) (PCL, see the structure in Figure 1(a)) of multiple molecular weights. The simulated systems are listed in Table 1. In order to describe the interatomic forces in the systems, two types of force fields were combined, the original OPLS-AA ^10^ and the modified L-OPLS ^11^. More specifically, L-OPLS was used as the main source of information, since this force field is based on the parameters of OPLS-AA but was optimized for long hydrocarbon chains, as well as for alcohols and esters ^11^. This optimization is in line with the objective of this work, namely, to provide a reliable and relatively simple model of PCL that could be extended to experimentally relevant time and length scales as well as to relevant molecular weights. In addition, it provides an improvement with respect to the previously published data, which relied on OPLS-AA for description of oligomeric PCL. The full set of parameters used here together with the corresponding source can be found elsewhere ^18^. All the simulations were performed by open-access simulation package Gromacs ^19^.

**Table 1.** Details about the systems under study. *M _w_* refers to the molecular weight of the chain, *N _c_* denotes the number of chains in the system, *N* is the number of monomers per chain, *ρ*_A_ refers to the density of the atomistic system and *ρ*_CG_ to the density of the coarse-grained system.

Label of the system	*N*	*M _w_* [g/mol]	*N _c_*	*ρ*_A_ [kg/m ^3^]	*ρ*_CG_ [kg/m ^3^]
PCL10	10	1143	70	928 ± 7	930±30
PCL30	30	3426	70	928 ± 4	940±20
PCL50	50	5709	70	928 ± 3	950±10
PCL100	100	11416	70	928 ± 2	939±9
PCL125	125	14270	70	928 ± 2	939±9

Due to the lack of data on PCL modelled by L-OPLS force field, we designed the methodology as follows. In the first stage, we simulated the PCL chains in water. This step in our methodology serves as a comparative analysis with prior research. A key consideration was the lack of available studies that investigate a range of PCL’s molecular weights in a molten state. Consequently, we opted to use solvent simulations to verify our model. The radius of gyration *R _g_* of these systems was then confronted with the published data, to corroborate the behaviour of our systems. The detailed description of the preparation of these systems, the full set of data and the results used for the comparison of *R _g_* with the literature data are publicly available ^18^. Briefly, after the preparation of a single chain molecule with the desired molecular weight (see Table 1), the chain was placed in vacuum to achieve a collapsed configuration. In continuation, so-obtained molecule was simulated in water at 300K. The results obtained from these simulations were highly satisfactory.

The initial state of systems in melt was composed of the partially collapsed chains (see the description in ref. 18 for more information about the preparation of these chains). 70 polymer chains were randomly placed in a simulation box, big enough to avoid overlaps. The density was then adjusted by performing a short simulation at 50 bar. This preparation process is similar to the process published in ref. 20 and is used to remove gaps between chains and obtain a homogeneous density. The systems were then equilibrated in a similar way as published in ref. 21. Briefly, the systems were first equilibrated at 600K, then they were cooled down to 500K and then equilibrated at this temperature. The length of the equilibration run at each temperature depended on the chain length and the radius of gyration was monitored as an indicator of the equilibrated structure. Before the production run, a run of 100 ns was performed applying the same conditions as the production run. Namely, the PME method was used for the calculation of the electrostatic interactions, the velocity rescaling with a stochastic term was applied to maintain the temperature at 500K and the Parrinello-Rahman barostat kept the pressure at 1 bar. The time step was 2 fs and the bonds containing hydrogens were constrained by the LINCS algorithm ^22^. The atomistic production run was 400 ns long for chain lengths of *N* = 10 − 100 and 600 ns long for PCL125. All runs, the equilibration runs and the productions runs, are publicly available ^23^. The coarse-grained simulations were performed at constant temperature of 500K and constant pressure of 1 bar, using Nosé-Hoover thermostat and the Parrinello-Rahman barostat. The time step was 5 fs and the bonds were constrained by the LINCS algorithm ^22^. The coarse-grained simulations are also publicly available ^24^.

## Results and Discussion

Polymer chains in melt typically exhibit dense and homogeneous packing, where monomers are isotropically surrounded (see also Figure 1(b)) and long-range intermolecular forces are effectively screened. This screening behavior in the melt state, where chains “screen” each other from long-range forces, causes the chain to statistically behave like an ideal chain without the excluded volume effect. This leads to a particular theoretical scaling of fundamental structural properties with the number of monomers in the chain and since in the validation step we focused on confronting the results from the atomistic model with systems in solution, in what follows we focus on confirming this theoretical assumption in melt for both atomistic and coarse-grained model derived systematically from the atomistic configurations.

In the first place, to characterize the polymer’s local structure, monomer distribution functions were calculated directly from the atomistic simulation data. These distributions, encompassing bond lengths, angles, dihedrals, and monomeric radial distribution functions, served as critical target data for the subsequent coarse-graining procedure. In this procedure, the atoms were grouped into “super-atoms” or beads. More specifically, in our case each bead represented one monomer, i.e., the internal beads of the chain consisted of 18 atoms and the terminal ones of 19 and 20, due to the presence of the carboxyl and hydroxyl groups at the extremes (see also Figure 1(a)). The centre of the bead was then placed in the centre of mass of each monomer. One example of such a coarse-graining procedure is shown in Figure 1(c).

**Figure 2.**
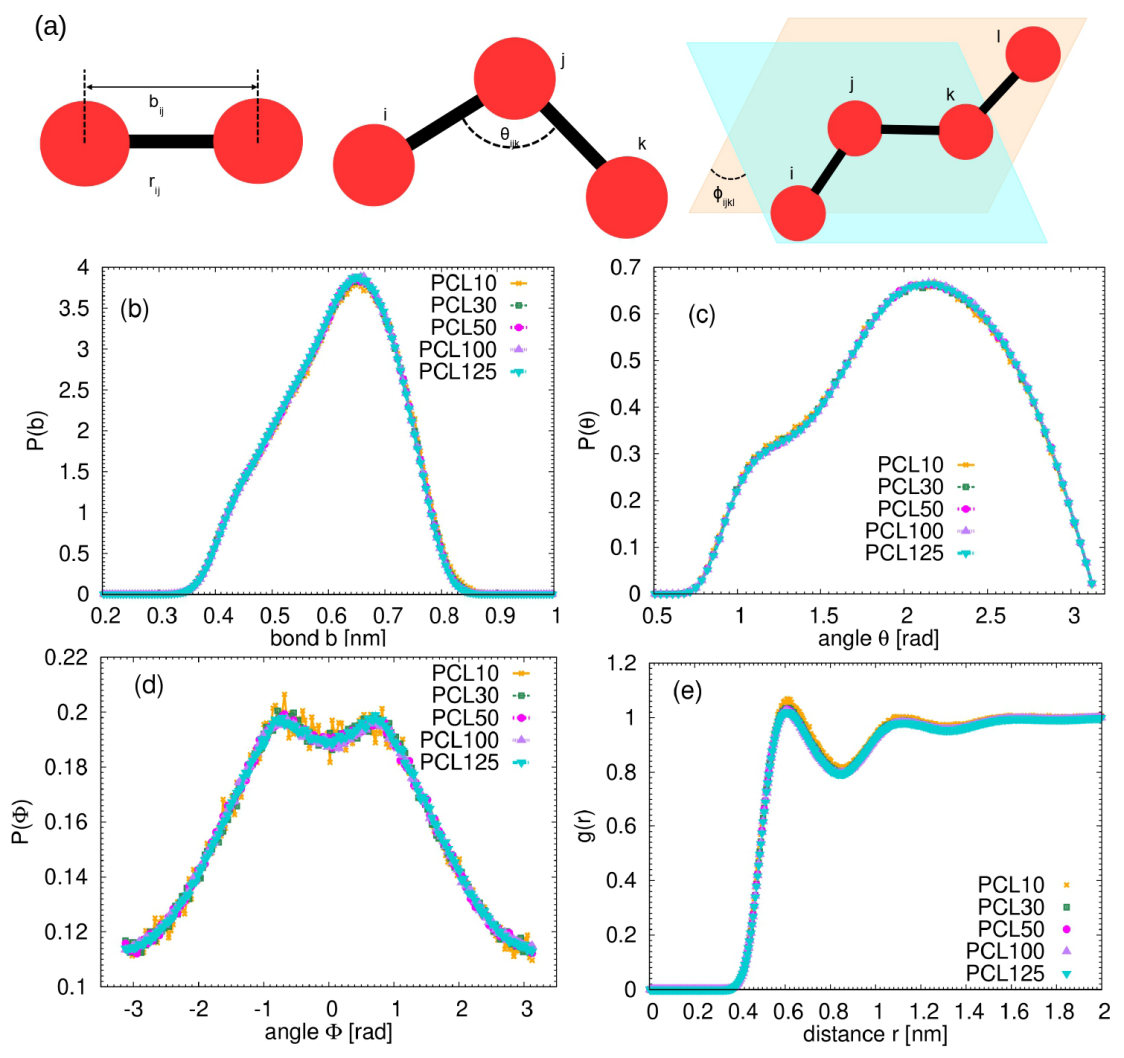
( **a**) Schematic illustration of the bonded interactions and the parameters plotted in the monomeric distributions ( **b**– **e**) calculated from the atomistic simulations. Each bead represents one single monomer. ( **b**) Distribution of the bond length between monomers. ( **c**) Distribution of the angles between monomers. ( **d**) Distribution of the dihedrals between monomers. ( **e**) Monomeric radial distribution function for the studied systems.

The monomer distribution functions measured from the atomistic simulations are shown in Figure 2 together with the sketch of the measured internal geometrical parameters.

Despite covering a wide range of molecular weights, no significant differences in these distributions across studied PCL chains were found. This observed consistency in local structural features, regardless of the overall chain length, implies that the immediate chemical environment and inherent conformational flexibility of the PCL monomer are largely independent of the macroscopic chain size in the melt. This independence significantly simplifies the development of coarse-grained models, as a potential derived from shorter oligomers can reliably be applied to longer chains for these specific local properties, forming a robust basis for bottom-up polymer modeling.

**Figure 3.**
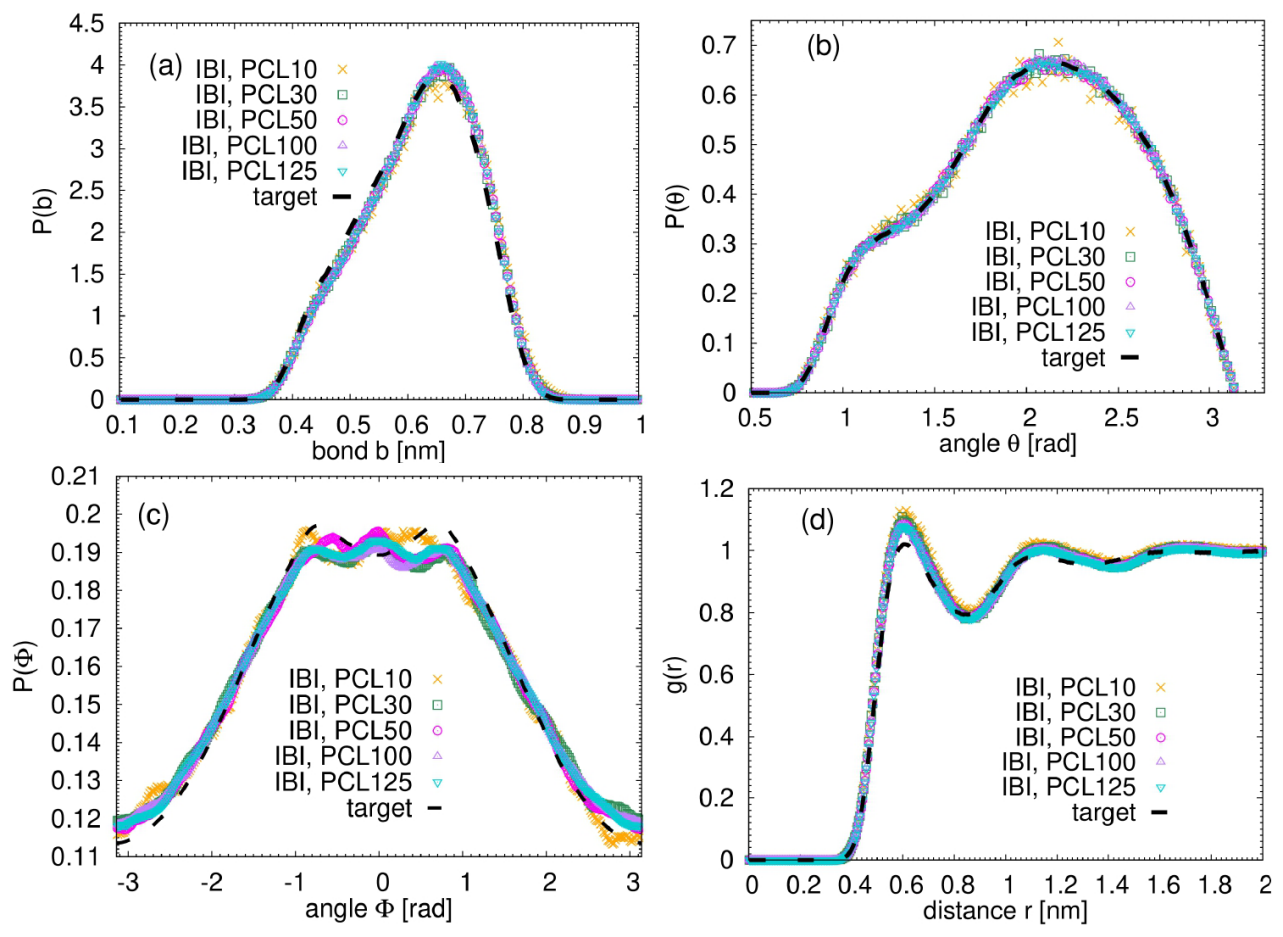
Results of the IBI procedure. Each bead represents one single monomer. ( **a**) Distribution of the bond length between monomers. ( **b**) Distribution of the angles between monomers. ( **c**) Distribution of the dihedrals between monomers. ( **d**) Monomeric radial distribution function for the studied systems. The dashed line represents the data from the atomistic simulation of PCL125.

In continuation, we applied Iterative Boltzmann Inversion (IBI), as one of the most powerful methods used to derive effective mesoscale potentials from atomistic simulations. IBI iteratively refines an initial potential guess until structural distribution functions match target data from detailed simulations ^9^. Figure 3 illustrates the results of the IBI procedure in comparison to the target atomistic data, which were chosen to be the data from the longest simulated polymers, PCL125. The notable agreement observed between the IBI curves and the target data across all distributions confirms that such a simple and versatile method is capable of accurately reproducing the structural characteristics derived from atomistic simulations throughout the entire studied molecular weight range.

Once ensuring that the sampled distributions achieved good agreement with the target data obtained from atomistic simulations, the derived potentials were subsequently utilized to simulate the coarse-grained (CG) systems. In what follows we present structural analysis of both atomistic and coarse-grained systems to demonstrate that our simple methodology renders consistent results at atomistic as well as at the mesoscale.

**Figure 4.**
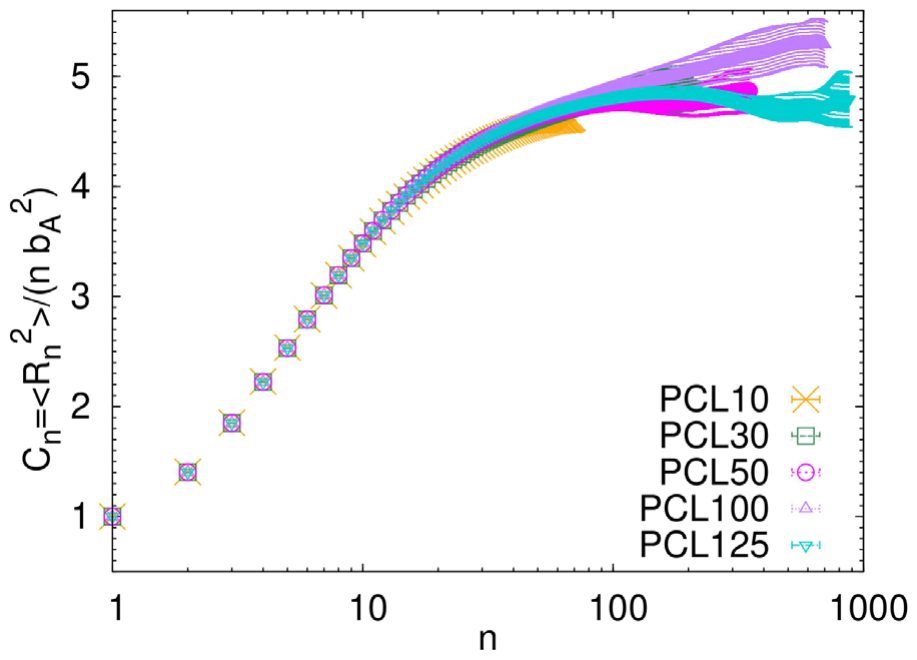
Characteristic ratio *C _n_* as a function of the distance between atoms along the backbone of the atomistic chains.

One important measure of the internal molecular structure is the plot of internal distances, *R _n_* ^2^ */n*, as a function of the separation *n* along the chain. When measured among pairs of atoms along the atomistic backbone, this quantity is directly related to the characteristic ratio *C _n_* (or *C*_∞_), which serves as a crucial measure of a polymer chain’s intrinsic flexibility or extension in its unperturbed state. The *C _n_* calculated directly from the atomistic simulations is shown in Figure 4. The characteristic ratio *C _n_* is defined as $C_{n}=\langle R_{n}^{2}\rangle /\left(nb_{\text{A}}^{2}\right),$ where $\langle R_{n}^{2}\rangle$ is the mean squared distance between atoms, *n* represents the separation (of the atoms) along the chain backbone, and *b*_A_ is the average bond length in the atomistic simulations. In Figure 4 the maximum separation equals to *n*_max_ = 7 *N* − 1, where *N* is the number of monomers in the chain. The value of *C _n_* changes with *n*. As *n* gets very large, *C _n_* approaches a maximum value, which we call *C*_∞_. The value of *C*_∞_ visually estimated from the plateau at long *n* in Figure 4 is *C*_∞_ ≈ 5. This estimated value is in good agreement with the previously published experimental results ( *C*_∞_ = 3.9−6 ^25– 28^) and with the theoretical estimation from the rotational isomeric state calculations ( *C*_∞_ = 4.1 − 4.5 ^29^).

**Figure 5.**
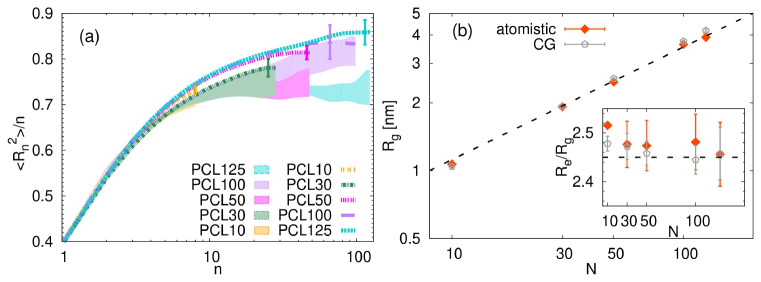
( **a**) Monomer internal distances as a function of the separation *n* between monomers, for the atomistic (shaded area) and CG systems (lines). The largest error bars for the CG systems are also plotted. ( **b**) Radius of gyration as a function of the number of monomers, *N*. The inset shows the ratio of the end-to-end distance, *R _e_*, to the radius of gyration, *R _g_*. The dashed line in both cases represent the theoretical prediction, namely *R _g_* ∼ *N* ^0.5^ and *R _e_/R _g_* = √6. The error bars were calculated by the standard block method, using 4 blocks of simulation data.

In Figure 5(a) the internal distances, *R _n_* ^2^ */n* are shown as a function of the separation *n* along the chain for studied PCL systems. Note that in this case, in order to be able to compare the atomistic and CG systems, *n* denotes the separation of the monomers (i.e., the difference in their indices) and not of the atoms. Giving the connection between the internal distances and the characteristic ratio, the differences in *R _n_* ^2^ */n* in Figure 5(a) reflect the differences in chain stiffness. As depicted in the figure, the value of *R _n_* ^2^ */n* generally increases with increasing separation *n*. For longer PCL chains, particularly those with a higher number of monomers (e.g., PCL100 and PCL125), the function demonstrates a tendency to reach a plateau at larger *n* values. For *n* equal to the number of monomers *N*, *R _n_* ^2^ represents the squared value of the average chain span, i.e., the end-to-end distance *R _e_* ^2^. The visual agreement between the atomistic and coarse-grained results in Figure 5(a) is particularly good for PCL10, and PCL30, which highlights the consistent representation of these internal distances across different simulation resolutions. For longer chains, the CG model seems to overestimate the chain stiffness, leading to higher values of the plateau at high values of *n*. This deviation of larger-scale dimensions is attributed to the fact, that while IBI performs very well at reproducing local structural correlations, it often struggles to accurately capture larger-scale, long-range properties of polymer chains. The phenomenon is not inherent to IBI and was reported in various studies dealing with the coarse graining ^8, 30^.

Figure 5(b) presents the radius of gyration *R _g_* as a function of the number of monomers *N* for both the atomistic and coarse-grained representations of PCL. Our analysis of the atomistic data for *R _g_* reveals a clear upward trend with increasing number of monomers, consistent with theoretical scaling predictions for polymers in the melt, namely *R _g_* ∼ *N* ^0.5^. This finding is important as it confirms that our atomistic models accurately capture the unperturbed conformational state of PCL in a dense environment.

When examining the coarse-grained results for *R _g_*, we observe that these values for chains with *N >* 30 are generally above those obtained from the atomistic simulations for the same chain lengths. This outcome is in line with slightly more stretched character of these CG chains in comparison to their atomistic analogues observed in Figure 5(a). Despite the quantitative differences, the CG *R _g_* values seem to maintain a similar scaling trend with *N* as their atomistic counterparts, indicating that the coarse-graining procedure successfully preserves the fundamental chain-length dependence of structural dimensions.

The inset of Figure 5 provides further insight into the conformational characteristics by illustrating the ratio of the end-to-end distance and the radius of gyration, *R _e_/R _g_*. For ideal chains, this ratio is theoretically *R _e_/R _g_* = *√6*. Our atomistic PCL chains in melt show *R _e_/R _g_* ratio that approaches this theoretical ideal value as chain length increases, more specifically, the theoretical value is reached for *N >* 30. The CG models also reflect this trend, although minor deviations from the atomistic data are present.

**Figure 6.**
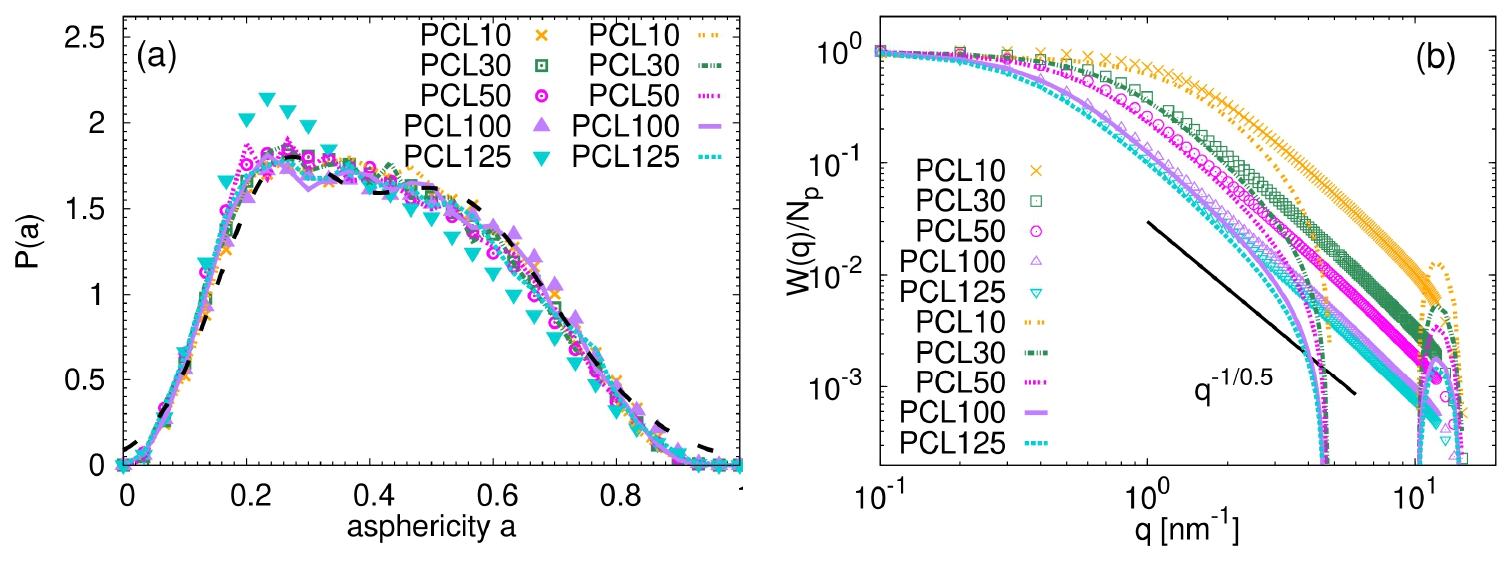
( **a**) Asphericity distribution for atomistic (points) and coarse-grained (lines) PCL systems. The dashed black line represents the theoretical expression reported in ref. 31 for unentangled poly(ethylene oxide). ( **b**) Form factor divided by the number of particles in the molecule as a function of the *q* vector for atomistic (points) and coarse-grained (lines) PCL systems. The solid line indicates the ideal scaling in the intermediate range of *q* values.

Figure 6(a) presents the asphericity distribution functions for the studied chains. The asphericity quantifies the shape of the molecules and is measured in simulation by calculating the geometrical inertia tensor:

$$T_{\alpha \beta }=\frac{1}{2N_{p}^{2}}\sum_{i}^{N_{p}}\sum_{j}^{N_{p}}(r_{i\alpha }-r_{j\alpha })(r_{i\beta }-r_{j\beta }),\;(1)$$

where *α,β* denote the Cartesian coordinates and *N _p_* represents the total number of particles in the chains, i.e., the number of atoms in the atomistic systems and *N _p_* = *N* for the CG systems. The eigenvalues of *T _αβ_*, labelled as *λ*_1_, *λ*_2_, *λ*_3_, are subsequently used to estimate the asphericity parameter from the following relation:

$$\;a=\frac{{\left(\lambda_{2}-\lambda_{1}\right)}^{2}+{\left(\lambda_{3}-\lambda_{1}\right)}^{2}+{\left(\lambda_{3}-\lambda_{2}\right)}^{2}}{2{\left(\lambda_{1}+\lambda_{2}+\lambda_{3}\right)}^{2}}.\;(2)$$

The more spherical the molecule, the closer its asphericity value is to 0. The distributions in Figure 6(a) for both atomistic and CG systems are identical within the obtained accuracy, with a sole exception of PCL125 system, which appears slightly more spherical at atomistic scale. The collected data have some features of the bimodal distributions expected by the theoretical model of a chain in *θ* conditions ^32^. The bimodal character originates from the two distinct molecular conformations present in *θ* state: a collapsed conformation with lower asphericity and a coil conformation. The distributions are in a perfect agreement with the data reported previously for other common unentangled synthetic polymers in melt (see the dashed line in Figure 6(a) and the Supplementary Information in ref. 33 which reports the unpublished data for refs. 8, 34 and data from ref. 31) and for poly(lactic acid) in melt ^33^, which confirms that the used model reproduces the expected behaviour of polymers in dense environments. It is important to note that, in contrast to poly(lactic acid) (PLA) ^33^, a related sustainable polymer, we found no evidence that the asphericity parameter depends on chain length, even with the shortest PCL10 chain.

To gain further insight into the internal molecular packing, the single-molecule form factor, *W*( *q*), was calculated. For a robust comparison between the coarse-grained and atomistic chains, *W*( *q*) was subsequently normalized by the number of particles *N _p_* per chain. The form factor probes the internal molecular packing at different characteristic length scales, represented by 1 */q*, where *q* is a wave vector. Focusing on the length scales larger than the bond length (i.e., ⟨ *b*_A_⟩ ≈ 0.148 nm for atomistic and ⟨ *b*_CG_⟩ ≈ 0.66 nm for CG systems) and lower than the radius of gyration, 1 */b* ≥ *q* ≥ 1 */R _g_*, data in Figure 6(b) show a perfect overlap between the CG and atomistic systems for the given chain length. In addition, within this *q* regime, a fractal regime *W*( *q*) ∼ *q* ^−1 */ν*^ is anticipated and confirmed. The determined exponent *ν* = 0.5 is typical for ideal Gaussian chains. In line with previous findings, this observation reinforces that the chains are following their predicted behavior. It is interesting to note, that a sudden drop of *W*( *q*) at *q* ≈ 3 nm ^−1^ and a consequent maximum at *q* ≈ 11 nm ^−1^ is a consequence of a spherical nature of the CG units (see e.g., ref. 35 for *W*( *q*) of a compact sphere).

As also described and demonstrated above, IBI method excels at reproducing the structural distributions between coarse-grained super-atoms, matching those obtained from atomistic simulations. However, CG models often exhibit faster dynamics compared to their atomistic counterparts, due to the implicit nature of friction and the averaged degrees of freedom. To accurately reproduce the dynamical properties, it is necessary to rescale the dynamics of the CG systems, often by applying a time scaling factor that compensates for the lower monomeric friction coefficient in the coarse-grained model ^36– 38^. In order to obtain this factor, we calculate two dynamical properties: the characteristic time related to the rotational movement of the chain and mean squared displacement of the center of mass.

The orientational autocorrelation function of the end-to-end vector for a chain provides a hint about the terminal dynamics of the chain, i.e., about the chain relaxation at long times. This function, denoted as *C*( *t*), quantifies the temporal correlation of the chain’s end-to-end vector orientation and is defined as:

$$\;C(t)=\frac{\vec{R_{e}}(t_{0}+t)\cdot \vec{R_{e}}(t_{0})}{\left|\vec{R_{e}}(t_{0}+t)∥\vec{R_{e}}(t_{0})\right|},\;(3)$$

where $\vec{R_{e}}(t+t_{0})$ is the end-to-end vector of the polymer chain at time *t*+ *t*_0_, and $\vec{R_{e}}(t_{0})$ is the end-to-end vector at the time origin *t*_0_. The angle brackets ⟨·⟩ denote an ensemble average.

**Figure 7.**
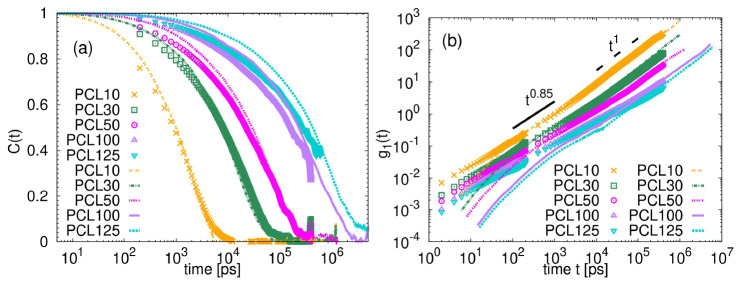
( **a**) Correlation function *C*( *t*) of the end-to-end vector as a function of time for all simulated systems. ( **b**) Mean squared displacement of the center of mass of the chains as a function of time. Lines correspond to the CG systems and points to the atomistic ones. The time *t* for the CG data in ( **a**, **b**) was multiplied by the shift factor *t _CG_* to match the atomistic data. The thick solid and dashed black lines in ( **b**) are guides for the eye and illustrate the power laws indicated above them.

The data on *C*( *t*) collected from the atomistic and CG simulations are show in Figure 7(a). Note that the autocorrelation functions for the CG chains are considerable slower than those of their atomistic analogues and that the time axis in the CG data was rescaled (see below).

Analysis of the decay of *C*( *t*) allows for the extraction of relaxation times that govern the overall reorientation of the chain. While theoretical models like the Rouse model describe the dynamics of unentangled, short polymers, predicting an exponential decay for its Rouse modes, real polymer melts often exhibit more complex features, such as stretched exponentials, due to factors like intramolecular bond correlations, excluded volume effects, and chain stiffness. Therefore, we fitted the data with the following function:

$$\;f(t)=A\exp(-{\left(t/\tau \right)}^{\beta })\;(4)$$

where *A* ≤ 1 is a prefactor, *τ* is the characteristic time (either *τ*_A_ or *τ*_CG_) and *β* is the stretch exponent. The results obtained from this analysis for the atomistic samples are summarized in Table 2. The same procedure was repeated for the CG samples, obtaining *τ*_CG_ and *β*_CG_. Comparing the characteristic times in Table 2, the faster dynamics in CG systems is corroborated. Comparing the stretch exponents and the data overlap in Figure 7, it is clear that the *C*( *t*) functions for CG chains exhibit behavior more akin to simple exponential decay, as it can be anticipated for models with reduced degree of details. Their systematically higher *β*_CG_ values, relative to the *β*_A_ values of comparable molecular weight chains, lead to noticeable deviations in the *C*( *t*) functions at early stages.

**Table 2.** Results of the analysis of the dynamical properties. *τ* and *β* refer to the characteristic time and stretch exponent obtained from the fit of the end-to-end correlation function. Parameters labelled with A were obtained from the atomistic simulations and those labelled with CG from the CG simulations. The error bars were calculated by the standard block method using 4 blocks of simulation data. *t _CG_* is the shift factor obtained as the ratio *t _CG_* = *τ*_A_ */* *τ*_CG_. Units labelled with * are units in CG simulations, which do not correspond to the physical units.

Label of the system	*τ*_A_ [ns]	*β*_A_	*τ*_CG_ [ns] *	*β*_CG_	*t*_CG_
PCL10	1.42±0.04	0.73±0.04	0.12±0.01	0.89±0.06	12±1
PCL30	13.5±0.7	0.65±0.04	1.1±0.3	0.67±0.05	12±3
PCL50	42±5	0.62±0.05	2.7±0.4	0.6±0.1	16±3
PCL100	350±110	0.48±0.06	12±3	0.55±0.08	29±14
PCL125	640±80	0.43±0.02	20±3.5	0.6±0.1	32±7

The scaling factor *t _CG_*, essential to map the CG data and to account for differences in the friction in both types of simulations, was calculated as *t _CG_* = *τ*_A_ */τ*_CG_. Note that after applying this scaling factor and rescaling the time axis in the *C*( *t*) time dependence, there is an apparent visual mismatch in CG and atomistic data for PCL100 and PCL125. We attribute this to the fact that by rescaling the data by *t*_CG_, i.e., by a ratio of characteristic times obtained from the stretched exponential function, the overlap should happen primary around the time scales where *C*( *t*) ≈ 1 */e*, thus around the characteristic time *τ*_A_. Unfortunately, data from the atomistic simulations in this given time window are noisy and the rescaled CG data are expected to show a good match with the atomistic systems at the time scales not reached in our simulations.

To describe the translational dynamics, we calculated the mean squared displacement of the center of mass, *g*_1_( *t*):

$$\;g_{1}(t)=\frac{1}{N_{c}}\sum_{i=1}^{N_{c}}\langle {\left|{\text{R}}_{\text{COM},i}\left(t_{0}+t\right)-{\text{R}}_{\text{COM},i}\left(t_{0}\right)\right|}^{2}\rangle \;(5)$$

where **R**_COM *,i*_( *t*_0_) is the position of the center of mass of the *i*th chain at time *t*_0_. The data for the atomistic systems and the rescaled data for the CG systems are shown in Figure 7(b). At intermediate time scales, before reaching the diffusive regime, *g*_1_ exhibits a sub-diffusive behavior very similar to the one reported for multiple unentangled systems, *g*_1_ ∼ *t* ^0.85^ ^39^. Detailed analysis of the origin of this sub-diffusive motion is beyond the scope of this work, however, as described in ref. 39, it indicates that unentangled polymers do not behave as ideal chains in a uniform medium. Instead, their dynamics are intrinsically linked to interactions with their polymeric environment, acting as a “fixed-point” effect on their dynamic properties ^39^. Concerning the agreement between the atomistic and rescaled coarse-grained *g*_1_( *t*)s, as the chain length increases the time window of the perfect overlap shrinks, and particularly, for PCL100 and PCL125, the data only match at the long time scales. This is in line with our previous observation about the expected agreement at terminal times scales, i.e., in the diffusive regimes, which are in the case of the longest chains beyond the simulation time window.

**Figure 8.**
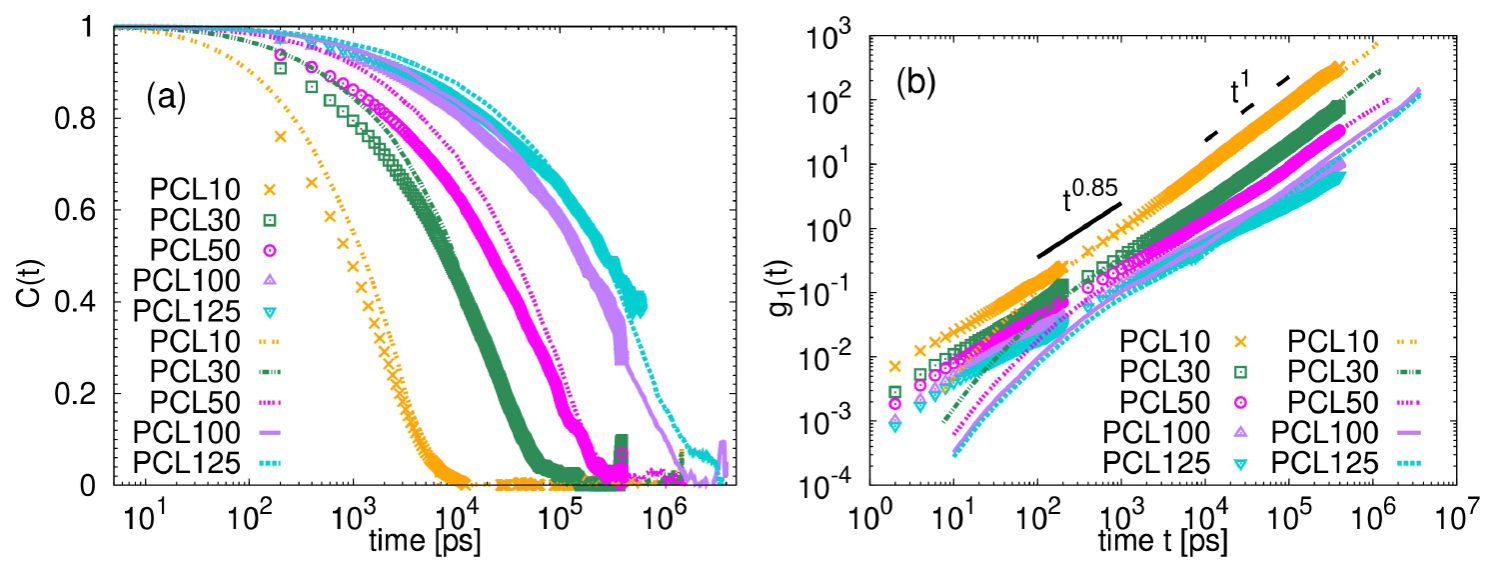
( **a**) Correlation function *C*( *t*) of the end-to-end vector as a function of time for all simulated systems. ( **b**) Mean squared displacement of the center of mass of the chains as a function of time. Lines correspond to the CG systems and points to the atomistic ones. The time *t* for the CG data in ( **a**, **b** was multiplied by the shift factor *t* ^MSD^_CG_ estimated from the onset of to the diffusive regime in the mean squared displacement. The thick solid and dashed black lines in ( **b**) are guides for the eye and illustrate the power laws indicated above them.

In order to provide an alternative estimation of *t*_CG_, we calculated the scaling factor from the *g*_1_ data. We chose a characteristic time for the center-of-mass diffusion, *t*_1_ as the time at which the chain diffuses its own size, i.e., at the time when *g*_1_( *t*_1_) = *R _g_* ^2^. Consequently, we estimated *t*_CG_ as the ratio of these characteristic times in the atomistic and in the CG simulations for the given chain length, i.e., *t* ^MSD^_CG_ = *t*_1 *,A*_/ *t*_1 *,CG*_. Since only the chains with *N <* 100 reached the diffusive regime, the estimated values of *t* ^MSD^_CG_ are 15, 15 and 20 for PCL10, PCL30 and PCL50. Assuming that the value of the scaling factor should reach a constant value at high molecular weights ^8^, we used *t* ^MSD^_CG_ = 20 for PCL100 and PCL125 to be able to plot the full set of data in Figure 8. Figure 8 shows that while this alternative estimation leads to a better apparent agreement in *g*_1_( *t*) for PCL10, PCL30 and PCL50 and at intermediate time scales also for PCL100 and PCL125, it overestimates the friction in rotational dynamics in CG systems with low molecular weight and *N <* 100 (see the *C*( *t*) functions in Figure 8(a)).

**Figure 9.**
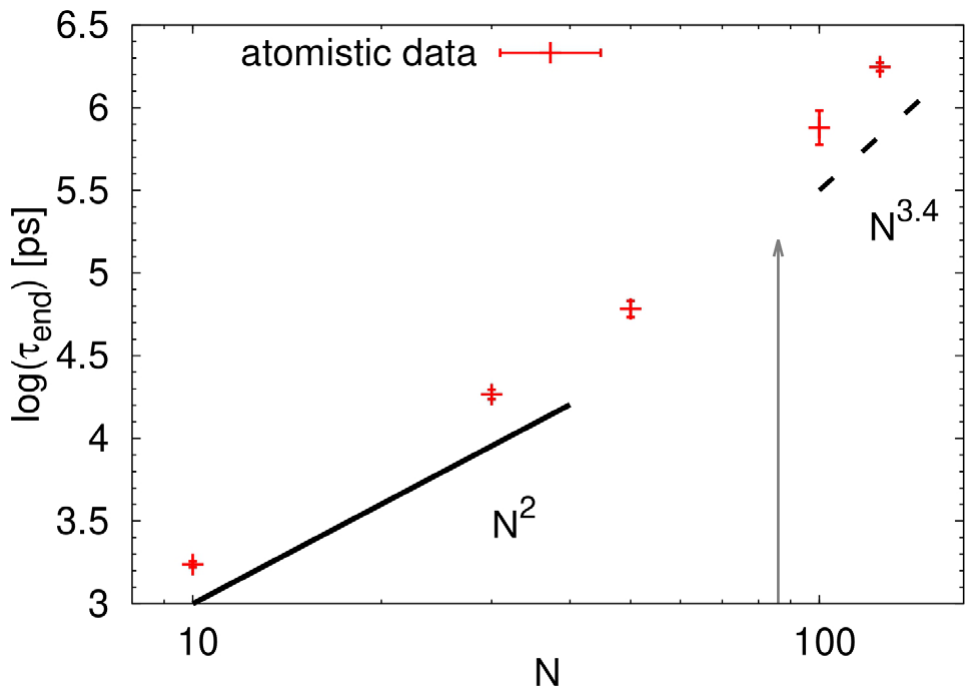
Terminal relaxation time *τ*_end_ as a function of the number of monomers *N*. The black lines are guides for the eye and indicate the scaling regimes for the Rouse model (solid line) and for the entangled regime (dashed line). The grey arrow points out to the value of 2 *N _e_* estimated from the packing length.

To provide a quantitative analysis of the dynamical properties, we calculated the terminal relaxation time using the data reported in Table 2 from the following relation:

$$\;\langle \tau_{\text{end}}\rangle =\frac{\tau_{\text{A}}}{\beta_{\text{A}}}\Gamma \left(\frac{1}{\beta_{\text{A}}}\right)\;(6)$$

where Γ denotes the gamma function. The data are plotted as a function of the number of monomers in Figure 9. Note that the terminal relaxation time obtained in this way can be related to the terminal relaxation in rheological measurements. In addition, the Rouse model predicts the scaling of *τ*_end_ ∼ *N* ^2^ for the unentangled linear polymers ^40^ while as the entangled regime is reached, a scaling of *τ*_end_ ∼ *N* ^3.4^ is anticipated ^41^. Looking at the illustrated power laws in Figure 9, it seems that the crossover between the Rouse regime and the entanglement regime occurs at chain lengths around 50 monomers. The critical molecular weight *M _c_*, which marks the onset to the entanglement regime, is usually a multiple of the entanglement molecular weight, *M _e_*, with *M _c_/M _e_* being between 2 and 7 ^42^. Having in mind the limited number of points in our study, the estimation of *M _e_*, or the number of monomers per entanglement strand *N _e_* is not directly possible from the plot in Figure 9.

As the estimation of crossover in Figure 9 is only visual, we proceed with a more rigorous way related to the structural property of a system called packing length, *p*. Packing length relates the size of the polymer, namely its end-to-end distance *R _e_*, to the density and the monomer mass *M _w_*:

$$\;p=\frac{M_{\text{w}}}{\langle R_{e}^{2}\rangle \rho N_{A}},\;(7)$$

where N _*A*_ denotes the Avogadro’s constant. Considering that in our systems the density is independent of the molecular weight (see Table 1) and the scaling of *R _e_* with *N* and thus with *M _w_* is ideal, the packing length does not exhibit chain length effects. Therefore, we averaged the obtained *p* over the studied chain lengths, which resulted in ⟨ *p*⟩ = 0.27 ± 0.01 nm. This value is close to the value of various synthetic polymers, such as polybutadiene ( *p* = 0.21 nm) and polyisoprene ( *p* = 0.31 nm) ^42^. In addition, is in between the values reported for experimental PCL systems, namely *p* = 0.166 nm in ref. 25 and *p* = 0.315 nm in ref. 43. It is interesting to note that the calculation of the packing length from experiments is usually done through the relation *G _N_* ∝ *p* ^−3^, where *G _N_* is the entanglement modulus ^25^. Therefore, even a small difference among published *G _N_* from the rheological experiments may cause a huge difference in the final value of *p*. Having estimated the value of *p* from simulations, we can calculate *M _e_* such as:

$$\;M_{e}=n_{t}^{2}\rho {\text{N}}_{A}p^{3}\;(8)$$

where *n _t_* is a dimensionless number with a value of approximately 21 ^42^. The resulted value equals to *M _e_* = 4850 g/mol, which results in *N _e_* ≈ 43. Assuming for simplicity that *M _c_* = 2 *M _e_*, the onset to the entanglement regime would lie around *N* ≈ 86 (see the arrow in Figure 9). The experimental values for *M _e_* of PCL range between 1600 g/mol to 3900 g/mol ^25, 44– 46^, with the latest reported value of 8626 g/mol ^43^. During the quantitative comparison, one should keep in mind that the experimental values were estimated from the rheological measurements and thus effects such as polydispersity, crystallization and temperature effects should be considered. Giving that our estimation of *M _e_* relies solely on static properties, the agreement between the reported values and the one obtained in this work is satisfactory. This agreement is corroborated by the visual transition observed in Figure 9.

## Conclusions

This study successfully developed and validated a systematic coarse-graining approach for poly( *ϵ*-caprolactone) (PCL) in the melt state, demonstrating its capability to quantitatively capture key structural and dynamical properties across a wide range of molecular weights.

Our atomistic simulations, combining the L-OPLS and OPLS-AA force field for a better affinity with the long chains, provided robust and consistent local structural features (bond lengths, angles, dihedrals, and radial distribution functions) that were largely independent of chain length. This consistency significantly simplified the coarse-graining process, allowing for the derivation of effective mesoscale potentials from shorter oligomers that could be reliably applied to longer chains. The Iterative Boltzmann Inversion (IBI) method proved highly effective in reproducing the structural characteristics from atomistic simulations across the entire studied molecular weight range, confirming its versatility and accuracy for local correlations.

Analysis of structural properties at both atomistic and coarse-grained levels revealed good agreement for internal distances, particularly for shorter chains (PCL10, PCL30). For longer chains, the coarse-grained model showed a tendency to overestimate chain stiffness and larger-scale dimensions, a known limitation of IBI in capturing long-range properties. Despite these deviations in chain stretching, the coarse-grained model successfully maintained the fundamental chain-length dependence of structural dimensions, as evidenced by the consistent scaling trend of the radius of gyration, *R _g_*, with the number of monomers, *N*. Both atomistic and coarse-grained *R _g_* values exhibited scaling consistent with theoretical predictions for ideal Gaussian chains in the melt ( *R _g_* ∼ *N* ^0.5^), indicating that the chains adopt an unperturbed configuration. Furthermore, the ratio of end-to-end distance to the radius of gyration ( *R _e_/R _g_*) for atomistic PCL chains approached the theoretical ideal value of √6 for chains with *N >* 30, a trend also reflected in the coarse-grained models. The analysis of conformational shapes, including asphericity distributions and form factors, also demonstrated good agreement between the atomistic and coarse-grained representations, further validating the structural accuracy of the coarse-grained model.

Regarding the dynamical properties, we presented results for orientational and translational dynamics of the entire chains. The coarse-grained model was appropriately time-scaled to account for the difference in the chain friction. Both dynamical properties, relaxation of the chain end-to-end vector and the mean squared displacement of the center of mass were used to extract the scaling factors. In addition, the dependence of the terminal relaxation time of the chains on the chain length was analysed, with the aim to shed light on the transition from the unentangled to entangled behavior. Using the packing length calculated from the atomistic simulations, we estimated the entanglement molecular weight to be around 4850 g/mol, which compares reasonable well with the reported experimental values estimated from the rheological spectra and with the visual inspection of the *N* dependence of the terminal relaxation times from the atomistic simulations.

In summary, this work provides a reliable and relatively simple systematic coarse-grained model for linear PCL, offering a valuable tool for extending computational studies to experimentally relevant time and length scales. While the coarse-grained model accurately reproduces local structural correlations and general scaling trends, further refinement may be beneficial for precise quantitative prediction of large-scale, long-range properties in very long chains. This methodology represents a significant step towards facilitating the development of computational approaches for tuning the properties of PCL-based materials, contributing to more sustainable material development. In addition, it may serve as a first step towards quantitative predictions of rheological properties of PCL-based systems with well-controlled composition and architecture. Taking advantage of the well-defined samples in simulations, the confrontation of the simulation results with the experimentally available data and theoretical models may lead to better understanding of the structure-properties-performance relationship, without having to deal with complex features present in experimental PCL samples such as self-nucleation ^44^.

## Ethics and consent

Ethical approval and consent were not required.

## Data Availability

The detailed description of the preparation of the atomistic systems, the code for the preparation of a chain with a desired number of monomers, the full set of data for the simulations in water and the results used for the comparison of *R _g_* with the literature data are publicly available ^18^ ( https://doi.org/10.5281/zenodo.17106011) All atomistic runs are available in Zenodo ( https://doi.org/10.5281/zenodo.16943800). The coarse-grained simulations with the corresponding tables for the potentials obtained from IBI procedure are also publicly available ( https://doi.org/10.5281/zenodo.16944321).
