# Supplementary material for: Development of a systematic coarse-grained model for poly(ε-caprolactone) in melt
Source: Open Res Eur. 2025 Dec 24;5:296. Originally published 2025 Sep 23. [Version 2] doi: 10.12688/openreseurope.21354.2 (PMC12521900; doi:10.12688/openreseurope.21354.2)
Supplement: Peer review history

# Reviewer report: Reviewer response for version 2

**DOI:** 10.21956/openreseurope.23913.r66862

**Published:** 2026-01-08

**Authors:** Sara Ranjbareslamloo

**Affiliations:** 1 The University of Toledo, Toledo, Ohio, USA

Approved

Is the study design appropriate and does the work have academic merit?

Yes

Is the work clearly and accurately presented and does it cite the current literature?

Yes

If applicable, is the statistical analysis and its interpretation appropriate?

Not applicable

Are all the source data underlying the results available to ensure full reproducibility?

Yes

Are the conclusions drawn adequately supported by the results?

Yes

Are sufficient details of methods and analysis provided to allow replication by others?

Yes

Reviewer Expertise:

Material Science Engineer/Mechanical Engineering focusing on multiscale computational modeling

I confirm that I have read this submission and believe that I have an appropriate level of expertise to confirm that it is of an acceptable scientific standard.

# Reviewer report: Reviewer response for version 1

**DOI:** 10.21956/openreseurope.23096.r61276

**Published:** 2025-10-17

**Authors:** Paola Carbone

**Affiliations:** 1 The University of Manchester, Manchester, England, UK

This is an interesting manuscript about the development of a coarse-grained (CG) model for poly ε-caprolactone (PCL) melts. PCL is an important polymer where a plethora of applications in particular in drug delivery but also in additive manifacturing. PCL is also difficult to simulate especially in solutions, so having a well tested CG model  is important.

 The paper is clear and the methodology well explained. The results show that the CG model works well  across a range of molecular weight. The quality and clarity of the figures and plots make the read smooth.

 I therefore suggest publication with just a minor revision which is the following

 1. For the initial testing of PCL in water, the authors might want to compare their results with the work https://doi.org/10.1021/jp505348t or  

https://doi.org/10.1002/cjce.23761 whre the OPLSAA force field was used.

Is the study design appropriate and does the work have academic merit?

Yes

Is the work clearly and accurately presented and does it cite the current literature?

Yes

If applicable, is the statistical analysis and its interpretation appropriate?

Yes

Are all the source data underlying the results available to ensure full reproducibility?

Yes

Are the conclusions drawn adequately supported by the results?

Yes

Are sufficient details of methods and analysis provided to allow replication by others?

Yes

Reviewer Expertise:

molecular simulations of polymers

I confirm that I have read this submission and believe that I have an appropriate level of expertise to confirm that it is of an acceptable scientific standard.

# Response

**Published:** 2025-12-03

**Authors:** Petra Bacova

We thank the Reviewer for a positive assessment of our manuscript. In the following we address the Reviewer’s comments and describe in detail the respective changes in the new version of the manuscript.  

 1) *For the initial testing of PCL in water, the authors might want to compare their results with the work https://doi.org/10.1021/jp505348t or https://doi.org/10.1002/cjce.23761 where the OPLSAA force field was used.* We thank the Reviewer for highlighting these studies.

**Response: **We have incorporated the data from these references into the manuscript section related to the verification of our model in aqueous conditions. Furthermore, we have added these references to the Introduction of the main manuscript.

# Reviewer report: Reviewer response for version 1

**DOI:** 10.21956/openreseurope.23096.r61279

**Published:** 2025-10-17

**Authors:** Sara Ranjbareslamloo

**Affiliations:** 1 The University of Toledo, Toledo, Ohio, USA

**Abstract and Introduction**

Use *poly(ε-caprolactone)* consistently, not *poly(caprolactone)*.
2. Abstract must include purpose, methods, key results, and significance (≤ 250 words). Current abstract meets length but lacks explicit mention of dataset DOIs.
In Section2 (Line 26-90) multiple sentences repeat context (e.g., biodegradable plastics, PCL sustainability). Combine to streamline flow. Rewrite lines 30–36 to one concise paragraph summarizing PCL relevance.
Add recent citations to strengthen background.
remove line-break hyphenation artifacts such as equili-brated, behav-iour.

**Methods and simulation details **

1. In Section 2, lines 30–33, the phrase “centers around” should be revised to “focuses on” for a more formal academic tone, and “The polymer melt behavior” should be corrected to “Polymer-melt behavior” to ensure proper compound noun usage and stylistic consistency.
2. Insert non-breaking space between number & unit (e.g., 600 K, 2 fs, 1 bar).
3. Eqs. (1–2) must italicize variables (e.g., Rg, τ, ρ). Subscripts should be upright when denoting constants.
4. Clarify whether bond/angle/dihedral distributions were directly sampled from AA MD or fitted via IBI (Line 59–61).
5. Thermostats/barostats: Justify difference between AA (velocity-rescale) and CG (Nosé–Hoover) schemes (Section 3, Line37–41).
6. Ensure equations numbered sequentially; currently Eq. (5) is cited before Eq. (4).
7.  “primary” → “primarily” in time-scale overlap sentence (Sec3).

**Results and Discussion **

1. In the result section; “Values exhibited scaling consistent…” → “Values exhibit scaling consistent…” (present tense for results)
2. In the result section; Ensure subject–verb agreement in “The data shows → show.”
3. Discuss *C∞* discrepancy (≈ 5) – note force-field sensitivity. (explain why your computed C∞ value differs from values reported in the literature and mention that C∞ can vary depending on the force field (the atomistic potential model used).
4. Explain CG density deviation vs AA results (Result Section, line 65–67)
5. Add paragraph on scalability/transferability for higher MW chains (> 2 Mₑ)
6. Include a small table comparing AA vs CG simulation time per ns per chain length

**Conclusions**

1. In conclusion, add one sentence specifying which time-scale mapping (τ_end vs τ_MSD) is recommended for future CG predictions.
2. In conclusion avoid repetition of “facilitate computational studies to optimize PCL materials” (appears twice). Merge into one impactful closing.

**References**

1. Unify author initials spacing (e.g., “Bačová P et al.” not “Bačová P.et.al.”).

Is the study design appropriate and does the work have academic merit?

Yes

Is the work clearly and accurately presented and does it cite the current literature?

Yes

If applicable, is the statistical analysis and its interpretation appropriate?

Not applicable

Are all the source data underlying the results available to ensure full reproducibility?

Yes

Are the conclusions drawn adequately supported by the results?

Yes

Are sufficient details of methods and analysis provided to allow replication by others?

Yes

Reviewer Expertise:

Material Science Engineer/Mechanical Engineering focusing on multiscale computational modeling

I confirm that I have read this submission and believe that I have an appropriate level of expertise to confirm that it is of an acceptable scientific standard, however I have significant reservations, as outlined above.

## References

[1] : Enhancing mechanical properties of PCL Biopolymers with APTES-Functionalized Bioactive Glass Nanoparticles: A molecular dynamics study. *Computational Materials Science* .2025;255: 10.1016/j.commatsci.2025.113930 10.1016/j.commatsci.2025.113930

# Response

**Published:** 2025-12-03

**Authors:** Petra Bacova

We thank the Reviewer for a positive assessment of our manuscript. Some comments appear to relate to the unpublished version or formatting of the manuscript. As these issues are not present in the latest online version, we have informed the Editorial office to ensure clarity regarding the most current document. In the following we address the Reviewer’s comments and describe in detail the respective changes in the new version of the manuscript.

**Abstract and Introduction**

*1) Use poly(ε-caprolactone) consistently, not poly(caprolactone).*

**Response: **The term was corrected.

 2) *Abstract must include purpose, methods, key results, and significance (≤ 250 words). Current abstract meets length but lacks explicit mention of dataset DOIs*.

**Response: **The dataset DOIs were omitted from the abstract in compliance with the journal's policy against including references in this section.

*3) In Section2 (Line 26-90) multiple sentences repeat context (e.g., biodegradable plastics, PCL sustainability). Combine to streamline flow. Rewrite lines 30–36 to one concise paragraph summarizing PCL relevance.*

**Response: **The introduction was rewritten. More specifically, we removed repeated statements in the paragraph describing PCL properties and we improved the state-of-the art section. *4) Add recent citations to strengthen background.* New citations were added. *5) Remove line-break hyphenation artifacts such as equili-brated, behav-iour.* The artifact is not present in the online version of the manuscript.

**Methods and simulation details**

*1) In Section 2, lines 30–33, the phrase “centers around” should be revised to “focuses on” for a more formal academic tone, and “The polymer melt behavior” should be corrected to “Polymer-melt behavior” to ensure proper compound noun usage and stylistic consistency.*

**Response: **The phrase “centers around” is not present in the online version of the manuscript. We kept the term “polymer melt behavior” to be consistent with the form used in most scientific and engineering contexts.

*2) Insert non-breaking space between number & unit (e.g., 600 K, 2 fs, 1 bar).*

**Response: **The error was corrected.

*3) Eqs. (1–2) must italicize variables (e.g., Rg, τ, ρ). Subscripts should be upright when denoting constants.*

**Response: **We applied these rules consistently to all equations throughout the manuscript to meet this academic standard.

*4) Clarify whether bond/angle/dihedral distributions were directly sampled from AA MD or fitted via IBI (Line 59–61).*

**Response: **The description of the IBI procedure was extended. The bond, angle, dihedral distributions as well as the radial distribution function were first calculated from the atomistic simulations (Figure 2 in the manuscript). Then the target atomistic distributions were converted into initial estimates of the potentials through direct Boltzmann inversion. In continuation, the IBI process was applied to refine the coarse-grained potentials.

*5) Thermostats/barostats: Justify difference between AA (velocity-rescale) and CG (Nosé–Hoover) schemes (Section 3, Line37–41).*

**Response: **Some of the atomistic systems were run on machines with GPUs, where the usage of Nosé-Hoover thermostat in Gromacs is discouraged. Therefore, we used temperature coupling with a velocity rescaling and a stochastic term, which ensures a proper ensemble sampling and is compatible with GPU-supported version of the simulation package. *6) Ensure equations numbered sequentially; currently Eq. (5) is cited before Eq. (4).*

**Response: **The artifact is not present in the online version of the manuscript.

*7) primary” → “primarily” in time-scale overlap sentence (Sec3).*

**Response: **The error was corrected.

**Results and Discussion**

*1) In the result section; “Values exhibited scaling consistent…” → “Values exhibit scaling consistent…” (present tense for results)*

**Response: **The sentence was corrected.

*2) In the result section; Ensure subject–verb agreement in “The data shows → show.”*

**Response: **The sentence was corrected.

*3) Discuss C∞ discrepancy (≈ 5) – note force-field sensitivity. (explain why your computed C∞ value differs from values reported in the literature and mention that C∞ can vary depending on the force field (the atomistic potential model used).*

**Response: **We agree with the Reviewer that the characteristic ratio may vary for different force fields. The value obtained from our simulations is in excellent agreement with the experimental data and in very good agreement with the values obtained from the Rotational Isomeric State (RIS) calculations, given the accuracy of the estimation and the limitations of the RIS model. The error bar apparent from Fig. 4 is around 0.5, so having this value in mind, the characteristic ratio would be 5+/-0.5, which agrees with the RIS estimation.

*4) Explain CG density deviation vs AA results (Result Section, line 65–67)*

**Response: **Even though there are some discrepancies in the average values (about 1%), for the vast majority of systems the coarse-grained densities fall within the calculated error bars of the atomistic data. A minor exception was observed only for the PCL50 system, which exhibited a slight deviation outside this range. Given the good agreement observed across the full set of molecular weights, we consider the systematic coarse-graining procedure to be successful in reproducing the target properties.

*5) Add paragraph on scalability/transferability for higher MW chains (> 2 Mₑ)*

**Response: **We added a text related to the scaling factor to clarify the issue.

*6) Include a small table comparing AA vs CG simulation time per ns per chain length.*

**Response: **We included a comparison of the performance of the both models for the longest simulated chain.

**Conclusions**

*1) In conclusion, add one sentence specifying which time-scale mapping (τ_end vs τ_MSD) is recommended for future CG predictions.*

**Response: **A sentence about the scaling factor was added into Conclusions.

*2) In conclusion avoid repetition of “facilitate computational studies to optimize PCL materials” (appears twice). Merge into one impactful closing.*

**Response: **The artifact is not present in the online version of the manuscript.

**References**

*1)***** *Unify author initials spacing (e.g., “Bačová P et al.” not “Bačová P.et.al.”).*

**Response: **The artifact is not present in the online version of the manuscript.

# Reviewer report: Reviewer response for version 1

**DOI:** 10.21956/openreseurope.23096.r61285

**Published:** 2025-10-14

**Authors:** Dimitrios G Tsalikis

**Affiliations:** 1 ETH Zurich Foundation, Zürich, Zurich, Switzerland

The authors utilize iterative Boltzmann inversion (IBI) to create a coarse-grained (CG) potential for polycaprolactone based on all-atom (AA) simulations. The MD simulation results using this CG potential align closely with the AA data, validating the method. The paper is well written, with a clear presentation of the findings. Overall, this research will engage the community; however, there are a few points, especially points 2-4, that need minor revision.

1.  I am somewhat concerned about the predicted density from the AA simulations. Although this isn't critical for this work, I would expect oligomers (PCL 10) to have a clearly smaller density than the marginally entangled (PCL 125) due to enhanced free volume from the chain ends. Unfortunately, I couldn't find relevant experimental data. Can the authors clarify this behavior?
2. Including a brief discussion of the steps taken to obtain the CG potentials would be helpful. This would make the IBI process more reproducible for the community and increase the manuscript’s visibility. For example, were all contributions tuned at the same time or one after the other, and how many refinement cycles were done for each contribution (bonds, angles, dihedrals, non-bonded)? What criteria did the authors utilize to stop refinement for each contribution?
3. Building on the previous point, during the IBI process, it is unclear whether intramolecular contributions were considered when calculating the monomeric radial distribution function. If they were, did the authors exclude first (or second or third) neighbours, since these interactions are likely captured by the bonded contributions? Is a similar exclusion applied to the corresponding CG potential?
4. Since this manuscript discusses an approach to enable community MD simulations of larger PCL chains (with molar mass> 2M _e_), which cannot be practically modelled with AA resolution due to computational cost, the authors need to demonstrate the transferability of the potential to higher MWs. This is straightforward for conformational properties. Regarding relaxation or diffusion dynamics, I understand that rescaling is necessary. But they could at least try to estimate τ ^MSD^_CG_ or at τ _AA_ through extrapolation, allowing calculations for higher MWs without conducting additional AA-MD runs.
5. And a minor point, to demonstrate to readers the power and potential of this work: I suggest the authors include a table quantifying the computational cost for AA simulations at least for the highest molar mass examined, and compare it with the cost of the same simulations using the CG potential. 
6. Lastly, another minor.  I would suggest updating the legends in Figures 5a, 6ab, and 7ab to clearly distinguish between AA-MD and CG-MD without mentioning the system name twice.                   

Is the study design appropriate and does the work have academic merit?

Yes

Is the work clearly and accurately presented and does it cite the current literature?

Yes

If applicable, is the statistical analysis and its interpretation appropriate?

Yes

Are all the source data underlying the results available to ensure full reproducibility?

Yes

Are the conclusions drawn adequately supported by the results?

Yes

Are sufficient details of methods and analysis provided to allow replication by others?

Partly

Reviewer Expertise:

Multiscale modeling of soft matter.

I confirm that I have read this submission and believe that I have an appropriate level of expertise to confirm that it is of an acceptable scientific standard.

# Response

**Published:** 2025-12-03

**Authors:** Petra Bacova

We thank the Reviewer for a positive assessment of our manuscript. In the following we address the Reviewer’s comments and describe in detail the respective changes in the new version of the manuscript.  

*1. I am somewhat concerned about the predicted density from the AA simulations. Although this isn't critical for this work, I would expect oligomers (PCL 10) to have a clearly smaller density than the marginally entangled (PCL 125) due to enhanced free volume from the chain ends. Unfortunately, I couldn't find relevant experimental data. Can the authors clarify this behavior?*

**Response: **We agree with the Reviewer, we also anticipated that the short PCL chain would exhibit lower density due to the chain end effects. However, the absence of this expected trend aligns with the lack of a molecular weight effect observed across the other static properties reported in this manuscript. Concerning the experimental studies, investigations of low molecular weight melt of poly(caprolactone) (PCL) are scarce, and the available PCL samples are typically semicrystalline, complicating direct comparison. However, one relevant study (10.1016/j.polymer.2020.122899) reported a slightly higher melt density for low molecular weight PCL samples compared to those with high molecular weight. This observation was attributed to the positive contribution of the ester linkers to the melt density, which they suggested was due to their associated steric hindrance.  

*2. Including a brief discussion of the steps taken to obtain the CG potentials would be helpful. This would make the IBI process more reproducible for the community and increase the manuscript’s visibility. For example, were all contributions tuned at the same time or one after the other, and how many refinement cycles were done for each contribution (bonds, angles, dihedrals, non-bonded)? What criteria did the authors utilize to stop refinement for each contribution?*

**Response: **The tuning was sequential, starting with the bonded interactions and then with the non-bonded. Before the pressure correction, all the contributions were also tuned at the same time. The convergence of the Iterative Boltzmann Inversion is quantified by defining a norm as the integral of the absolute difference between the target and the current step's unnormalized distributions. To ensure consistent scaling across all components, each distribution is first normalized such that the target distribution's integral equals unity. The overall convergence norm is then calculated as the mean of the norms from all distinct contributing potentials. IBI iterations stops when this mean norm drops below a set tolerance, or when the improvement between successive steps falls below a specified small percentage. We extended the text related to the IBI procedure correspondingly.  

*3.Building on the previous point, during the IBI process, it is unclear whether intramolecular contributions were considered when calculating the monomeric radial distribution function. If they were, did the authors exclude first (or second or third) neighbours, since these interactions are likely captured by the bonded contributions? Is a similar exclusion applied to the corresponding CG potential?*

**Response: **Considering the calculation of the potentials, the algorithm excludes short-range intramolecular contributions when calculating the non-bonded radial distribution function, since those short-range interactions are intended to be described by the bonded potentials. More specifically, the exclusion is defined by the number of bonds separating two CG atoms. In our case, the exclusion was applied up to 3 bonds away from each other. We added this information to the manuscript.  

*4. Since this manuscript discusses an approach to enable community MD simulations of larger PCL chains (with molar mass> 2Me), which cannot be practically modelled with AA resolution due to computational cost, the authors need to demonstrate the transferability of the potential to higher MWs. This is straightforward for conformational properties. Regarding relaxation or diffusion dynamics, I understand that rescaling is necessary. But they could at least try to estimate τ ^MSD^_CG_ or at τ _AA_ through extrapolation, allowing calculations for higher MWs without conducting additional AA-MD runs.*

**Response: **We agree with the Reviewer on the importance of demonstrating the transferability of our potentials to higher molecular weights. We have indeed addressed the transferability for conformational properties in the manuscript. Regarding the dynamics, while rescaling is necessary, we demonstrated that the rescaling parameter for the dynamics converges for long chains. Based on previous studies, we expect this parameter to be very close to the reported value for MWs beyond the range explicitly simulated, and thus, it should not change significantly for even longer chains. We have added a comment to the manuscript to explain this convergence.  

*5. And a minor point, to demonstrate to readers the power and potential of this work: I suggest the authors include a table quantifying the computational cost for AA simulations at least for the highest molar mass examined, and compare it with the cost of the same simulations using the CG potential?*

**Response: **We added a comparison of the computational cost for one representative system.  

*6. Lastly, another minor.  I would suggest updating the legends in Figures 5a, 6ab, and 7ab to clearly distinguish between AA-MD and CG-MD without mentioning the system name twice.*

**Response: **We modified the figures correspondingly.

# Reviewer report: Reviewer response for version 1

**DOI:** 10.21956/openreseurope.23096.r61280

**Published:** 2025-10-03

**Authors:** Tejas Shah

**Affiliations:** 1 The University of Texas at Dallas, Richardson, Texas, USA; 2 Biochemistry and Molecular Biology, Michigan State University (Ringgold ID: 3078), East Lansing, Michigan, USA

The manuscript presents coarse-grained modeling of poly(caprolactone) (PCL) polymers to predict melt properties using a bottom-up approach with all-atom simulations and iterative Boltzmann inversion (IBI) for coarse-graining. The topic is timely and relevant for the polymer modeling community. The manuscript can be considered for publication after the following points are addressed:

1. The overall writing is good. However, the introduction section would benefit from polishing. Some sentences are missing context, while certain statements are repeated and redundant. The authors are encouraged to refine this section to improve clarity and readability.
2. Comparison with Reported All-Atom Studies
  - Several all-atom simulation studies on PCL have been reported with shorter chains (10.1039/D4TB02789B, 10.1021/acs.jpcb.4c05368, 10.1016/j.cap.2018.07.011, 10.1021/acs.macromol.5c00158, 10.1080/07391102.2018.1541762). Did the authors compare their results with these works?
  - Since CG models often deviate from experimental values due to the absence of detailed atomistic interactions, how did the authors address this limitation in their parameterization?
  - If the purpose of CG was to access larger chain lengths, did the authors also perform simulations of higher molecular weight PCLs to test the robustness of the model?
3. If the entire monomer was mapped as a single bead, how were polarization effects and hydrogen-bond interactions from polar atoms accounted for in the CG model? Please clarify whether effective interactions were adjusted for this purpose.
4. The authors should clearly define how the monomer-to-monomer bond lengths, angles, and dihedrals were calculated. Were these distributions directly obtained from AA simulations, or were they imposed during the IBI process?
5. The reported CG densities show deviations compared to the all-atom model, even though the parameters were derived from AA simulations. Could the authors explain why this discrepancy occurs and what might be the source of such variation?

Is the study design appropriate and does the work have academic merit?

Yes

Is the work clearly and accurately presented and does it cite the current literature?

Yes

If applicable, is the statistical analysis and its interpretation appropriate?

Not applicable

Are all the source data underlying the results available to ensure full reproducibility?

Yes

Are the conclusions drawn adequately supported by the results?

Yes

Are sufficient details of methods and analysis provided to allow replication by others?

Yes

Reviewer Expertise:

Molecular Dynamics Simulations, Force Field Development, Polymer Chemistry, Biochemistry

I confirm that I have read this submission and believe that I have an appropriate level of expertise to confirm that it is of an acceptable scientific standard.

# Response

**Published:** 2025-12-03

**Authors:** Petra Bacova

We thank the Reviewer for a positive assessment of our manuscript. In the following we address the Reviewer’s comments and describe in detail the respective changes in the new version of the manuscript.

1. *The overall writing is good. However, the introduction section would benefit from polishing. Some sentences are missing context, while certain statements are repeated and redundant. The authors are encouraged to refine this section to improve clarity and readability*.

We modified Introduction as requested. More specifically, we removed repeated statements in the paragraph describing PCL properties and we improved the state-of-the art section.

1. *Comparison with Reported All-Atom Studies*

*-Several all-atom simulation studies on PCL have been reported with shorter chains (10.1039/D4TB02789B, 10.1021/acs.jpcb.4c05368, 10.1016/j.cap.2018.07.011, 10.1021/acs.macromol.5c00158, 10.1080/07391102.2018.1541762). Did the authors compare their results with these works?* We appreciate the reviewer's input and the suggested literature. However, because those works address copolymer chains and polymer blends, their findings are not directly applicable or comparable to the behavior of the homopolymer systems investigated in our manuscript. *-Since CG models often deviate from experimental values due to the absence of detailed atomistic interactions, how did the authors address this limitation in their parameterization?* We understand the Reviewer's concern regarding potential deviations from experimental values in coarse-grained (CG) models. We addressed this limitation by implementing a systematic parameterization approach where the CG interaction potentials were derived directly from high-quality atomistic simulations. This rigorous methodology guarantees the CG model inherently reproduces the structural properties (i.e., structural fidelity) of the reference atomistic system, ensuring the underlying physics is preserved. The reliability of our CG results stems from the accuracy of the atomistic model, which utilizes the advanced force field capable of quantitatively describing the system's properties. Our atomistic model has been thoroughly validated against existing simulation literature. We note that a direct, quantitative comparison to experimental data is not generally feasible for our specific system (monodisperse amorphous PCL at the studied molecular weight) because available experimental results are often biased by factors like crystallinity and polydispersity. However, for properties where a qualitative or semi-quantitative comparison is appropriate despite these limitations, we did include experimental data: specifically, we compare our results with rheological measurements in the final section of the manuscript. As noted in that section, this comparison is made with a discussion of the above-mentioned limitations. *-If the purpose of CG was to access larger chain lengths, did the authors also perform simulations of higher molecular weight PCLs to test the robustness of the model?* As confirmed by Figure 9, the longest polymer chain investigated in our work approaches the limit of the entangled regime. A thorough, quantitative study of fully entangled systems requires a substantially more intensive equilibration process and greater computational resources. Consequently, the detailed analysis of the dynamics within the fully entangled regime is beyond the scope of the current article and will be the focus of future work.  

1. *If the entire monomer was mapped as a single bead, how were polarization effects and hydrogen-bond interactions from polar atoms accounted for in the CG model? Please clarify whether effective interactions were adjusted for this purpose.*

Given the inherent simplifications of our coarse-grained model, polarization effects were not explicitly included. Regarding hydrogen bonds, the simulation temperature is high and the potential donor groups are present solely at the polymer chain ends. Because these conditions severely limit the formation and stability of H-bonds, making their effect minor, we judged their explicit inclusion unnecessary for accurate structural reproduction and thus omitted them from the coarse-grained representation. Nevertheless, for systems studied at lower temperatures, additional effective CG interactions accounting for hydrogen bonding could be incorporated, and this will be explored in future work.  

1. *The authors should clearly define how the monomer-to-monomer bond lengths, angles, and dihedrals were calculated. Were these distributions directly obtained from AA simulations, or were they imposed during the IBI process?*

We extended the discussion related to the IBI procedure. The bond, angle, dihedral distributions as well as the radial distribution function were first calculated from the atomistic simulations (Figure 2 in the manuscript). Then the target atomistic distributions were converted into initial estimates of the potentials through direct Boltzmann inversion. In continuation, the IBI process was applied to refine the coarse-grained potentials.  

1. *The reported CG densities show deviations compared to the all-atom model, even though the parameters were derived from AA simulations. Could the authors explain why this discrepancy occurs and what might be the source of such variation?*

Even though there are some discrepancies in the average values (only around 1%), for the vast majority of systems the coarse-grained densities fall within the calculated error bars of the atomistic data. A minor exception was observed only for the PCL50 system, which exhibited a slight deviation outside this range. Given the good agreement observed across the full set of molecular weights, we consider the systematic coarse-graining procedure to be successful in reproducing the target properties.
